# Partial Cholecystectomy Safe and Effective

**DOI:** 10.1155/1993/52802

**Published:** 1993

**Authors:** Md. Ibrarullah, L. K. Kacker, S. S. Sikora, R. Saxena, V. K. Kapoor, S. P. Kaushik

**Affiliations:** 1 Department of Surgical Gastroenterology Sanjay Gandhi Postgraduate Institute of Medical Sciences Lucknow India; 2 Department of Surgical Gastroenterology Sanjay Gandhi PostGraduate Institute of Medical Sciences Post Box # 375 Lucknow 226001 India

## Abstract

Patients undergoing surgical treatment for calculous disease were considered to have had a partial cholecystectomy performed when a part of the gall bladder wall was retained for technical reasons. Forty patients underwent partial cholecystectomy: for chronic cholecystitis (20), acute cholecystitis (4), Mirizzi's syndrome (14), portal hypertension or partially accesible gall bladder (one patient each). Four patients (10%) developed infective complications and two patients had retained common bile duct stones. In a mean follow up period of 13 months (range 1–36 mths), only 3 patients have ongoing mild dyspeptic symptoms while the rest have remained asymptomatic. Partial cholecystectomy has been found to be a safe and effective procedure in difficult cholecystectomy situations, since it combines the merits of cholecystectomy and cholecystostomy.


*HPB Surgery*, 1993, Vol. 7, pp. 61-65
Reprints available directly from the publisher
Photocopying permitted by license only

© 1993 Harwood Academic Publishers GmbH
Printed in the United States of America

### CASE REPORT
### PARTIAL CHOLECYSTECTOMY - SAFE AND EFFECTIVE

MD. IBRARULLAH, L.K. KACKER, S.S. SIKORA, R. SAXENA, V.K. KAPOOR and S.P. KAUSHIK

*Department of Surgical Gastroenterology, Sanjay Gandhi Postgraduate Institute of Medical Sciences, Lucknow, India*

Patients undergoing surgical treatment for calculous disease were considered to have had a partial cholecystectomy performed when a part of the gall bladder wall was retained for technical reasons. Forty patients underwent partial cholecystectomy: for chronic cholecystitis (20), acute cholecystitis (4), Mirizzi's syndrome (14), portal hypertension or partially accesible gall bladder (one patient each). Four patients (10%) developed infective complications and two patients had retained common bile duct stones. In a mean follow up period of 13 months (range 1–36 mths), only 3 patients have ongoing mild dyspeptic symptoms while the rest have remained asymptomatic. Partial cholecystectomy has been found to be a safe and effective procedure in difficult cholecystectomy situations, since it combines the merits of cholecystectomy and cholecystostomy.

KEY WORDS: Cholecystectomy, partial, subtotal, post cholecystectomy syndrome

### INTRODUCTION

Cholecystectomy is the most commonly performed operation the world over. Although the risk of operative injuries during cholecystectomy is reported to be 0.2 to 0.3 percent[1], the actual number of cases with such injuries becomes quite significant. The risk of injury is particularly high if there is anomalous anatomy and/or obliteration of Calot's triangle. The presence of portal hypertension or a partially intrahepatic small fibrosed gall bladder (GB) may cause alarming bleeding during dissection and thus may further increase the risk of such mishaps. Cholecystostomy under these circumstances has been suggested as a safer alternative[2,3]. However the results of cholecystostomy in terms of residual and recurrent stones[4,5] make it less attractive. The present study highlights the indications, conduct and outcome of partial cholecystectomy in these difficult situations.

### MATERIAL AND METHODS

The decision to perform a partial cholecystectomy was influenced by the presence of dense fibrosis and/or acute inflammation in the Calot's triangle, or suspicion of Mirizzi's syndrome.

Address correspondence to: Prof. S.P. Kaushik, Department of Surgical Gastroenterology, Sanjay Gandhi PostGraduate Institute of Medical Sciences, Post Box # 375, Lucknow 226001, India.

Forty patients underwent partial cholecystectomy between January 1989 and March 1992. The indications in these forty patients are shown in Table 1. Male to female ratio was 1:4. The mean age was 42 years (range 17–65 years) and the mean duration of symptoms was 51 months (range 2–264 months). Preoperative diagnosis was made on the basis of ultrasonography and/or oral cholecystography. Endoscopic retrograde cholangiography (ERC) or per operative cholangiography (POC) were performed selectively based on the presence or history of jaundice, raised serum alkaline phosphatase and/or suspicion or demonstration of stone on USG in addition to difficult anatomy. ERC was available in 13 patients and cholecystocholangiogram in one. The later patient had undergone cholecystostomy elsewhere for obliterated anatomy due to Mirizzi's syndrome type II. POC was performed in 8 patients. Difficult anatomy alone was however not an indication for POC. Associated pathology and the operative procedures performed are summarised in Tables 2 and 3 respectively.

**Table 1** Indications of partial cholecystectomy in forty patients

| | |
|---|---|
| Chronic cholecystitis | 20 |
| Mirizzi's syndrome | |
| Type I | 3 |
| Type II | 11 |
| Acute cholecystitis | 4 |
| Portal Hypertension | 1 |
| Partially accessible GB* | 1 |

*Severe kyphosis.

**Table 2** Associated pathology in forty patients

| | |
|---|---|
| Choledocholithiasis | 11 |
| Cholecystoduodenal fistula | 7 |
| Cholecystogastric fistula | 2 |
| Duodenal ulcer | 2 |
| Peri cholecystic abscess | 1 |
| Ruptured liver abscess | 1 |

### OPERATIVE TECHNIQUE

The GB was approached from the fundus and gently dissected from its bed upto the junction of neck and Hartman's pouch. It was opened at the fundus and its contents evacuated. The cholecystotomy was extended upto the Hartman's pouch and the mucosal aspect inspected for presence of any growth. The dissected wall of the GB was excised with a diathermy knife leaving behind Hartman's pouch adherent to Calot's triangle. The posterior wall of the GB in the presence of portal hypertension or when found to be densely adherent to the liver bed or partially embedded in

**Table 3** Operative procedures performed and the associated indications

| *Procedure* | *Indications* | *No. of patients* |
|---|---|---|
| Partial cholecystectomy | | |
| i) Retained Hartman's pouch only | Ch. Cholecystitis | 12 |
| | Ac. Cholecystitis | 02 |
| | Mirizzi's Type I | 03 |
| ii) Retained posterior wall | Ch. Cholecystitis | 8 |
| | Ac. Cholecystitis | 2 |
| | PH | 1 |
| | Inaccessible GB | 1 |
| Choledochoplasty and T-tube choledochostomy | Mirizzi's Type II +/− Choledocholithiasis | 10 |
| T-tube choledochostomy only | Choledocholithiasis | 1 |
| Choledochoduodenostomy | Choledocholithiasis | 6 |
| Highly Selective Vagotomy (HSV) | Associated DU | 2 |
| Cholecysto choledochoduodenostomy | Mirizzi's Type II | 1 |

PH = Portal Hypertension; DU = Duodenal ulcer the liver was left behind, usually in continuity with Hartman's pouch[6–8]. The cystic duct opening if identified was closed by a purse string suture of synthetic absorbable material (polygalactin) from the inside. The free margin of the pouch when feasible was approximated by a continuous absorbable suture.

In cases of Mirizzi's syndrome Type I (extrinsic compression of CBD) partial cholecystectomy was performed as described above. In the presence of Mirizzi's syndrome Type II (cholecysto-biliary fistula) flaps of GB wall were preserved for reconstruction of the CBD (choledochoplasty) around a T-tube or a bilioenteric anastomosis was performed as indicated[9,10]. Intraoperative cholangiogram was performed selectively by cannulating the cystic duct or by direct needle puncture of CBD. Additional procedures like CBD exploration or bilioenteric anastomosis were performed when indicated (Table 3). Drainage of the subhepatic space was done selectively.

### RESULTS

Four patients (10%) developed infective complications in the form of a wound infection (2) or intra abdominal abscess (2). The abscess was managed by ultrasound guided percutaneous catheter drainage in one, while in the other patient it drained spontaneously through the main wound. Retained CBD stones were seen in two patients. These were managed by endoscopic papillotomy and extraction in one and surgery in another patient. The average hospital stay was 12 days. There was no mortality in the series.

In a mean follow up period of 13 months (range 1–36 months) all patients except three are asymptomatic. The three patients with retained Hartman's pouch continue to have mild dyspeptic complaints.

### DISCUSSION

Partial cholecystectomy, has proved to be a safe and effective[6–8,11,12] option in patients with gall stone disease, where insistence on cholecystectomy may result in iatrogenic complications. It was required in about 8% of patients undergoing surgery for gallstone disease in our series. The sceptics however view this procedure as incomplete with a potential for long term morbidity. The possible disadvantages are — an increased incidence of infection (since the GB is opened early in the course of surgery), residual or recurrent stone formation in the GB remnant, and the risk of malignancy arising in the retained GB mucosa.

Four (10%) of our patients had infective complications. Similar observations have been made by others[6,7,12] and the infection rate is no greater than that reported after routine cholecystectomy and CBD exploration with or without bilioenteric anastomosis[13].

The risk of residual stones in the GB remnant after partial cholecystectomy is bound to be less than after cholecystostomy. The GB is opened under direct vision and all its contents are evacuated. Recurrence of stone, however, remains a possibility particularly if a blind pouch is formed by approximation of Hartman's pouch. This could also be obviated by virtually eliminating the dead space by close approximation. In our own experience so far no such recurrence has taken place. However a longer follow up would be required to draw any firm conclusions.

There are a few anecdotal reports of malignancy arising in GB after cholecystostomy[14,15] and in the cystic duct remnant after cholecystectomy[16]. The cause and effect relationship of carcinoma of the GB with stone disease is however yet to be proven beyond doubt, and the incidence of malignancy is not expected to be greater than the risk in the general population. A remote chance of developing malignancy should therefore, not deter a surgeon from performing a safe, effective and definitive surgical procedure in a given situation rather than insist on one which has a significantly increased chance of morbidity and possible mortality in a given high risk situation.

In conclusion, partial cholecystectomy appears to combine the merits of cholecystectomy and the safety of cholecystostomy. It should be routinely performed when the going is "tough" such as in cases with severe acute inflammation, frozen Calot's triangle due to chronic inflammation and in Mirizzi's syndrome.

### *References*

1. Kune, G.A. and Sali, A. (1981) Benign biliary stricture. In: *The practice of biliary surgery.* 2nd Edn. Blackwell Scientific Publications. Oxford
2. Glenn, F. (1981) Surgical management of acute cholecystitis in patients 65 years of age or older. *Ann. Surg.* **193**, 56–59
3. Aranha, G.V. (1983) Reply to selected summary: Cholecystectomy in cirrhotic patients. *Gasteroenterol.* **84**, 882
4. Welch, J.P. and Malt, R.A. (1972) Outcome of cholecystostomy. *Surg. Gynecol. Obstet.*, **135**, 717–721

5. Norrby, S. and Schonebeck, J. (1970) Long term results with cholecystolithotomy. *Acta Chir. Scand.*, **136**, 711
6. Bornman, P.C. and Terblanche, J. (1985) Subtotal cholecystectomy: for the difficult gall bladder in portal hypertension and cholecystitis. *Surgery*, **98**, 1–6
7. Cottier, D.J., McKay, C. and Anderson, J.R. (1991) Subtotal cholecystectomy. *Br. J. Surg.*, **78**, 1326–1328
8. Douglas, P.R. and Ham, J.M. (1990) Partial Cholecystectomy. *Aust N Z Surg.*, **60**, 595–597
9. Csendes, A., Diaz, C.J., Burdiles, P., Maluenda, F. and Nava, O. (1989) Mirizzi Syndrome and cholecystobiliary fistula: a unifying classification. *Br. J. Surg.*, **76**, 1139–1143
10. Baer, H.U., Mathews, J.B., Schweizer, W.P., Gertsch, P. and Blumgart, L.H. (1990) Management of the Mirizzi Syndrome and the surgical implications of cholecysto choledochal fistula. *Br. J. Surg.*, **77**, 743–745
11. Bickel, A., Lunsky, I., Mizrahi, S. and Stamler, B. (1990) Modified subtotal cholecystectomy for high risk patients. *CJS*, **33**, 13–14
12. Schein, M. (1991) Partial cholecystectomy in the emergency treatment of acute cholecystitis in the compromised patient. *J.R. Coll. Surg. Edinb.*, **36**, 295–297
13. Keighley, M.R.B., Drysdale, R.B., Quoraishi, A.H., Burdon, D.W. and Alexander Williams J. (1975) Antibiotic treatment in biliary sepsis. *Surg. Clin. North Am.*, **55**, 1379–1390
14. Kune, G.A. (1971) Carcinoma of Gall bladder 24 years after cholecystostomy. *Med. J. Aust.*, **1**, 544–545
15. So, C.B., Gibney, R.G. and Scudamore, C.H. (1990) Carcinoma of the Gall bladder: A risk associated with gall bladder preserving treatment for cholelithiasis. *Radiology*, **174**, 127–130
16. Carotenuto, F. and Simi, M. (1974) Carcinomatous papilloma of the cystic duct stump, report of a case. *Surgery in Italy*. **314**, 253–256

*(Accepted by S. Bengmark, 24 November 1992)*

### INVITED COMMENTARY

This paper adds further support for the technique of leaving the posterior wall of the gallbladder attached to its liver bed in the presence of severe pericholecystic inflammation and in patients with portal hypertension. Several recent reports have confirmed the ease and safety with which partial cholecystectomy can be performed in these difficult situations while achieving the same objectives as with the standard operation. The initial concern of an increased incidence of sepsis and continued mucous discharge from the residual gallbladder wall has not materialised nor the potential complications of leaving a long cystic stump. This paper also emphasises its use in cases with the Mirizzi syndrome where the risk of a "wedge type" injury to the bile duct is particularly high when dissecting Hartmann's pouch in Calot's triangle. The risk of a bile leak from injury to superficial bile ducts in the liver bed is also avoided in cases with severe peri-cholecystic inflammation.

Securing the cystic duct sometimes poses a technical problem and results in a bile leak when using the purse-string technique. However, with some care, the cystic duct orifice can be circumcised from within to achieve an adequate stump for closure. The more liberal use of this operation is recommended for the difficult gallbladder, particularly in the hands of less experienced biliary surgeons.

Professor P.C. Bornman
Professor of Surgery and Head Surgical Gastroenterology
University of Cape Town, South Africa

